# Supplementary material for: Maternal pandemic-related stress during pregnancy associates with infants’ socio-cognitive development at 12 months: A longitudinal multi-centric study
Source: PLoS One. 2023 Apr 17;18(4):e0284578. doi: 10.1371/journal.pone.0284578 (PMC10109481; doi:10.1371/journal.pone.0284578)
Supplement: S1 File — (DOCX) [file pone.0284578.s001.docx]

**Supplementary material**

**Table 1.** Characteristics of participants followed at 12 months (n= 91) and participants who dropped out from the study (n=222)

|  | | | | |  |  |  |  |  |  |  |  |  |
| --- | --- | --- | --- | --- | --- | --- | --- | --- | --- | --- | --- | --- | --- |
|  | **Study Sample at 12 months (n= 91)** | | | |  | **Participants who dropped out (n= 222)** | | | |  | **Groups comparison** | | |
|  | **Mean** | **SD** | **Minimum** | **Maximum** |  | **Mean** | **SD** | **Minimum** | **Maximum** |  | ***t*** | **gdl** | **p** |
| Gestational age (weeks) | 39.90 | 1.05 | 37.00 | 42.00 |  | 39.54 | 1.09 | 36.00 | 42.00 |  | -2.68 | 318 | .008 |
| Birth weight (grams) | 3382.59 | 421.42 | 2480.00 | 4435.00 |  | 3322.83 | 432.46 | 2050.00 | 4725.00 |  | -1.12 | 318 | .262 |
| Head circumference (cm) | 34.25 | 1.31 | 30.00 | 39.00 |  | 34.22 | 1.14 | 29.50 | 38.00 |  | -0.18 | 316 | .856 |
| Neonatal length (cm) | 50.59 | 2.01 | 46.00 | 56.00 |  | 50.15 | 1.89 | 45.00 | 56.00 |  | -1.85 | 316 | .065 |
| Apgar at minute 5 | 9.88 | 0.36 | 8.00 | 10.00 |  | 9.87 | 0.37 | 8.00 | 10.00 |  | -0.23 | 317 | .816 |
| Maternal age (years) | 33.15 | 4.68 | 18.00 | 46.00 |  | 33.39 | 4.43 | 21.00 | 51.00 |  | -0.42 | 316 | .676 |
| Maternal education (years of study) | 15.86 | 2.91 | 8.00 | 22.0 |  | 15.00 | 3.35 | 8.00 | 22.00 |  | -2.25 | 189.67 | .025 |
| Pandemic-related stress | 2.56 | 0.69 | 1.00 | 4.16 |  | 2.52 | 0.71 | 1.00 | 5.00 |  | -0.49 | 318 | .625 |
