# Supplementary material for: Maternal pandemic-related stress during pregnancy associates with infants’ socio-cognitive development at 12 months: A longitudinal multi-centric study
Source: PLoS One. 2023 Apr 17;18(4):e0284578. doi: 10.1371/journal.pone.0284578 (PMC10109481; doi:10.1371/journal.pone.0284578)
Supplement: Attachment — Submitted filename: PlosOne review (Final).docx [file pone.0284578.s002.docx]

In this study, the authors have assessed possible effects of COVID related prenatal maternal stress on infant socio-cognitive development. They found a significant negative linear association between maternal stress and the Infant’s socio-cognitive score at 12 months and conclude that findings suggest preventive measures should be taken during the pandemic. The authors are to be congratulated on a very interesting paper. The study is well-designed, the manuscript is well-written and the findings add important evidence about the impact of COVID on human health. This paper fits the scope, and meets the criteria for publication in Plos One and in my opinion should be accepted. I do have some suggestions that I think could make the paper even more interesting.

**Introduction:** The still-face is a well-validated paradigm designed to measure how an infant responds to a maternal stressor. The author’s have introduced a novel auditory stimulus during the still-face segment, coded orienting behaviors of the infant, and interpreted the results according to accepted orienting theory, which focuses on the cortically controlled orienting “response” phenomenon. The author’s might want to cite a recent paper that extensively reviews the orienting reflex theory, from Pavlov to present (Ludwig and Welch 2022 Frontiers in Psych) and concludes by identifying a novel autonomic socioemotional “reflex” (ASR) pathway. To that point, a recent still-face study coded orienting as a socioemotional reflex behavior pertaining to the mother-infant “relationship” - validating a new mother-infant *emotional connection* construct (see Hane AA et al. 2016 Act Paediatr).

**Discussion**: The authors might want to refer to other studies that showed that maternal stress before, during and after pregnancy, whatever the cause, has profound effects on the development and lifelong function of the infant's neurocognitive development. (Sharma etal 2022 Clin Epigenetics). The authors might want to cite some studies that looked at perinatal stress that emerged after 9-11. (Levendosky et al 2021 BMJ Open) ( Van Sjelghem etal 2022 Early Hum Dev), which are similar to those emerging from Pandemic stress.

The authors note in Discussion; “*The effect was specifically evident for infants’ tendency to produce active communications (e.g., waving by, smiling, vocalizing) toward an auditory stimulus while interacting with their mother*.” This observation deserves a paragraph to consider possible explanations of the orienting reflex data in this study. For instance, ASR theory predicts that those infants emotionally connected to the mother will display approach behaviors (i.e., attend to, orient to, etc.), while those not emotionally connected would display lessor or avoidant orienting behaviors. While mother-infant connection was not assessed in this study, it would be interesting in future studies to test the ASR hypothesis by conducting post-hoc analyses of mother-infant video interactions collected in this study using the Welch Emotional Connection Screen (WECS) to assess infant orienting to the mother and their emotional connection. Going forward, should the authors do further longitudinal follow-up studies, autonomic measures, such as HR and vagal tone could provide clinically relevant information from this study.
