# Supplementary material for: Maternal pandemic-related stress during pregnancy associates with infants’ socio-cognitive development at 12 months: A longitudinal multi-centric study
Source: PLoS One. 2023 Apr 17;18(4):e0284578. doi: 10.1371/journal.pone.0284578 (PMC10109481; doi:10.1371/journal.pone.0284578)
Supplement: Attachment — Submitted filename: R&R covid provenzi.docx [file pone.0284578.s003.docx]

The present study evaluates the associations between prenatal maternal stress during the pandemic and infant socio-cognitive functioning at 12-months postpartum. The authors find that infants of mothers who reported higher pandemic-related stress during pregnancy were less likely to point and demonstrate communication when presented with auditory stimuli. The study is clearly written, and has the potential to make a valuable contribution to the literature.

However, several points should be addressed. Overall, I take issue with the analytic approach (t-tests and correlations) and the cherry picking of results (frequency of socio-cognitive behaviors are not reported or interpreted, only the presence of them). Critically, the content of the pandemic stress measure is not presently available (the manuscript says it is available in the Supplement, but I do not have access to it). My recommendation for publication relies heavily on the availability of these items, since it is not a validated scale.

**Intro**

- The authors are conflating depression, anxiety, and stress – clinical symptoms of mental health disorders are different from perceived stress levels.

**Method**

- Need more information about how the domains of socio-cognitive functioning were coded. Were there multiple coders, and how was reliability assessed?
- The authors mention there was “remarkable sample attrition,” thus they only report results on infants with complete data. What was the original sample size, and what are the patterns of missing data? It is likely that large amounts of attrition will bias results. If possible, the authors should also consider using some method of imputation to reduce bias.
- Limitations of the remote interaction task should be considered. Are there any other published studies that have coded a remote parent-child interaction task? This is a novel method, and any evidence linking remote recordings to in-person recordings should be cited. If not, this is an important limitation to acknowledge, as the home environment is different from a lab environment (and, there might be connectivity issues with coding interactions remotely, etc).
- Additionally, I would appreciate more details on the remote interaction (overall quality of the recordings, percent of recordings that were unusable, instructions given to the parent, etc) as this may be of use to future researchers

**Results**

- An eligibility criteria for the study required mothers to test negative for COVID at delivery. The authors cite important evidence by Shuffrey et al, which reports neurodevelopmental delays of infants born to mothers who reported a COVID infection during pregnancy. Therefore, the authors should conduct a robustness check on mothers who tested positive for COVID during pregnancy with infant SCS scores (frequency & rate)
- The authors also collected data on not just the occurrence, but also the frequency by which infants displayed these pointing/communication behaviors during the auditory task. These results are missing from the manuscript
- The authors test for correlations between potential confounds but do not report the results of these tests. Why not just control for confounds using linear regression? Even if the covariates are not significantly related to either the predictor or outcome variable, they are theoretically important to the outcome and accounting for them will result in a more precise estimate. I recommend the authors use logistic regression for the dichotomous indicators to obtain odds ratios (and linear regression with standardized predictor variables for the continuous outcomes), and include covariates for the demographic characteristics listed
- Did you account for age of the infant at time of the assessment? This should also be included as a covariate, as there might be differences in developmental timing of pointing/communication abilities by age
- What is the socioeconomic spread of the sample? Maternal education is reported, but is there any further information such as income?
- In observing Figure 2, there appears to be an outlier (high PC-COM score). Are these results robust to outliers? This should be reported in the supplement, given the small sample

**Discussion:**

- The discussion begins with the sentence “the present study is among the first to suggest the presence of a long-lasting **effect** of maternal stress…”. This terminology is again used in the next paragraph “…socio-cognitive behaviors appeared to be at least partially **affected** by maternal stress exposure during pregnancy.” This implies causation.
- This study might be helpful to cite in either the intro or discussion – it is not correlational, which provides more compelling evidence: Imboden, A., Sobczak, B. K., & Griffin, V. (2021). The impact of the COVID-19 pandemic on infant and toddler development. *Journal of the American Association of Nurse Practitioners*. <https://doi.org/10.1097/JXX.0000000000000653>
- This recent study also is a natural experiment: Sperber, J. F., Hart, E. R., Troller-Renfree, S. V., Watts, T. W., & Noble, K. G. (2022). The effect of the COVID-19 pandemic on infant development and maternal mental health in the first 2 years of life. *Infancy*. <https://doi.org/10.1111/infa.12511>
- I challenge the authors to remove terminology such as “suboptimal” development, as it assigns value judgements to developmental trajectories. It is possible (and there is a large body of literature arguing this) that developmental differences may be “suboptimal” according to traditional Western contexts, but may be adaptive and *advantageous* in adverse contexts. I encourage the writers to remove value judgements of developmental trajectories, and explore this idea further – how might socio-cognitive difference due to early stress exposure be adaptive in certain contexts?
