# Supplementary material for: Maternal pandemic-related stress during pregnancy associates with infants’ socio-cognitive development at 12 months: A longitudinal multi-centric study
Source: PLoS One. 2023 Apr 17;18(4):e0284578. doi: 10.1371/journal.pone.0284578 (PMC10109481; doi:10.1371/journal.pone.0284578)
Supplement: Attachment — Submitted filename: pone-d-22-23782-r2_replies.docx [file pone.0284578.s005.docx]

**Article ID:** PONE-D-22-23782R1

**Title:** Maternal pandemic-related stress during pregnancy associates with infants’ socio-cognitive development at 12 months: A longitudinal multi-centric study

**Replies to comments from reviewers**

**Reviewer #2**

1. The authors did an excellent job responding to reviewer concerns. The current manuscript is well-written, presents compelling evidence, and will make a valuable contribution to the literature. I am happy to recommend this paper for publication.

**Reply.** Thanks for appreciating our revision.

2. A few small notes: Though statistically significant differences were found in maternal education and gestational age between those who attritted from the study and those who participated in the 12-month assessment, the magnitude of this difference is small (and ultimately, inconsequential). I think the authors should acknowledge this and present it as such using the mean differences as evidence. Rather, the bigger concern is that there are unobserved difference between the hundreds of dyads that attritted from the study and the follow-up sample (this is quite likely). I request the authors raise this concern in the limitation section, rather than the statistically significant differences found in the t-test.

**Reply.** Thanks for this suggestion, we have modified the text as follows:

*“Additionally, potential systematic patterns of missingness limit generalizability of the current findings. In particular, while small differences in gestational age and maternal education were found among dyads who attended the follow-up and dyads who withdrew, only variables collected at birth were available for comparison among the two groups, thus possible selective attrition on unobserved variables remained an issue.”*

3. Small typo found on the first line of page 11: “thought” should be “though”

**Reply.** Thank you again for careful reading of our manuscript. We have fixed the typo.

4. Figure 4 is a bit overwhelming to the eye. I recommend adding sub-titles above each scatterplot regarding which infant outcome is being represented (rather than only depending on the Y-axis) and limiting only 2 infant outcomes to a page. Additionally, I recommend putting the standardized coefficient and p-value from the regression analysis under each figure to contextualize the figure for the reader. Also specify for all figures when variables are standardized.

**Reply.** We agree with Rev #2. Figure 4 has been split into two new figures: the new Fig 4 reports the scatterplots for ORI, POI, COM; the new Fig 5 reports the scatterplot for the cumulative SCS. Statistics are reported in the text – anyway, they are also replicated in the Table legend now. These legends also highlight that all outcome variables on the y-axis are standardized.

**Reviewer #3**

This is a highly interesting and relevant manuscript relating pandemic related stress to socio-cognitive development in 12 month old infants. Many of the comments raised in the previous version have been well addressed. As I did not perform the previous review I have a few relatively minor comments.

**Reply.** Thanks for appreciating our work.

1. The authors argue that they found a "fairly linear or dose response relationship...", however, the regression models that they ran did not really test whether the relationships are linear. To do this the investigators would need to evaluate either a quadratic term for the PRS measure or perform spline analyses. This is important because the shape of the dose response curve matters when policy makers are considering the availability of 'formal and informal' support (although such support is always good). Indeed, inspecting figure 4 leads me to conjecture that (a) for some of the outcomes such as SCS and PC-ORI the relationships are not linear and (b) the relationships may differ for male and female infants.

**Reply.** We thank the Reviewer for making this point. It should be highlighted that the sentence “fairly linear or dose response relationship” was not referred to our findings, but to previous research – nonetheless, it has now been removed to avoid misinterpretations. At the same time, non-linearity of the observed associations was examined by the inclusion of a quadratic term for PRS within the regression models. Results for a quadratic effect of PRS on the study outcomes are as follows:

- No significant effect for ORI (as for linear testing)
- No significant effect for POI (as for linear testing)
- A tendency to statistical significancy for COM (R-squared = .10, p = .059; while the PRS linear term yielded a significant effect)
- A significant effect for SCS (R-squared = .09, p = .027; the linear effect had R-squared = .10, p = .017).

As such, we did not change our analytical plan. Nonetheless, adding the comparison between the linear and the quadratic association was not within the aims of the paper and we do not traced in previous literature enough support to make specific hypotheses on the shape of the linear or curvilinear association. Consistently, we opted to report on this linear association as it was tested as linear – of course, this does not rule out that in larger populations or in different settings the association might be curvilinear. For instance, few studies provided evidence that the association between maternal prenatal stress and children’s outcomes might be U-shaped, with mild to moderate levels of maternal distress during pregnancy actually promoting child development in healthy samples and being associated with more optimal outcomes, as compared to too little or too much prenatal maternal stress (e.g., Fernandes et al., 2014; DiPietro et al., 2006; Davis et al., 2017).

2. The large degree of missing data (ie loss to follow up) is troubling, especially since follow up is related to important confounders such as maternal education and gestational age. This could be better handled by performing inverse probability weighting which would account for some of the potential bias due to differential loss to follow up.

**Reply.** We thank the Reviewer for raising this issue. We agree that sample attrition is actually a significant issue in the current sample and is probably related to the pandemic time when recruitment occurs that limited participants’ engagement by allowing only remote data collection. Unfortunately, we only have information collected at birth about the dyads who withdrew from the study and, as also highlighted by Reviewer 2, while small differences were detected in terms of gestational age and maternal education among participants and not-participants to the follow-up phases, a number of unobserved differences are likely to exist, thus making our sample not representative of all observations. Thus, we do not feel appropriate to use inverse probability weighing in the current sample, as its effectiveness in “correcting” the selection bias highly depends on the availability of enough information, for the entire population, to predict the non-missingness probability (Oris et al., 2016; Narduzzi et al., 2014).

Following also comments from Reviewer 2, this has now been better acknowledged in the limitation section.

Oris, M., Roberts, C., Joye, D., & Stähli, M. E. (2016). Surveying human vulnerabilities across the life course: Balancing substantive and methodological challenges. In M. Oris, C. Roberts, D. Joye, & M. E. Stähli (Eds.), Surveying human vulnerabilities across the life course (pp. 1–25). Springer International Publishing. [https://doi.org/10.1007/978-3-319-24157-9_1](https://psycnet.apa.org/doi/10.1007/978-3-319-24157-9_1)

Narduzzi, S., Golini, M. N., Porta, D., Stafoggia, M., & Forastiere, F. (2014). L'uso dell'Inverse probability weighting (IPW) nella valutazione e "correzione" del selection bias [Inverse probability weighting (IPW) for evaluating and "correcting" selection bias]. *Epidemiologia e prevenzione*, *38*(5), 335–341.

3. Please use more descriptive headers for the tables. For example, in tables 1 and 2 should say something about the sample size, and whether this was an exploratory or confirmatory PCA. Table 3 should also have the sample size and indicate if this is the larger sample or the analytic sample. further this table should give the data to evaluate the loss to follow up, i.e. compare those who were followed at 12 months to those who were not.

**Reply.** Sample size has now been reported in all Table headers. Additionally, sociodemographic characteristics of the sample of dyads who dropped out from the study as well as differences with the study sample has now been summarized in Supplementary Table 1.

4. Please indicate whether successive steps include the variables in the previous step. That would make sense, but it is not clear the way the table is formatted.

**Reply.** Yes, the reviewer is correct. This has been added in the text.

5. Because the scores on the socio-cognitive development measures were not normally distributed there is a chance that extreme outliers may influence the results. Did the authors also consider the log transform of these variables, which would normalize the distribution.

**Reply.** This is a relevant issue. We indeed discussed about the possibility or opportunity to normalize the distribution. Nonetheless, it should be considered that these measures refer to specific precursors of socio-cognitive development that are emerging at the age (12-month) at which they were tested in this study. As such, it is expected that their distribution would be asymmetric as they are, reflecting the fact that some infants have already “mastered” these skills, while others are moving toward the same achievement. The distribution of these measures is consistent with this expectation. Indeed, we report here the asymmetry and kurtosis indexes for the socio-cognitive development measures:

| Measure | Asymmetry | Kurtosis |
| --- | --- | --- |
| ORI | 0.41 | -0.27 |
| POI | 2.26 | 4.94 |
| COM | 2.21 | 7.18 |
| SCS | 1.21 | 1.44 |

If we log-transform these measures, we obtain:

| Measure | Asymmetry | Kurtosis |
| --- | --- | --- |
| ORI | 0.18 | -0.37 |
| POI | 2.04 | 3.84 |
| COM | 1.72 | 4.27 |
| SCS | 0.95 | 0.78 |

These indexes are still problematic for some of the measures. Anyway, we ran the analyses using the log-transformed measures and we obtained comparable findings (we report here the final regression step):

- ORI: statistically non-significant
- COM: adjusted R-squared = .10, p = .042; PRS coefficient = -2.06, p = .042
- POI: statistically non-significant
- SCS: adjusted R-squared = .10, p = .016; PRS coefficient = -2.45, p = .016

In the light of these findings, we opted not to change our manuscript introducing the log-transformation.
